# Comparative immunogenicity analysis of intradermal versus intramuscular immunization with a recombinant human adenovirus type 5 vaccine against Ebola virus

**DOI:** 10.3389/fimmu.2022.963049

**Published:** 2022-08-31

**Authors:** Zhe Zhang, ZhengHao Zhao, Yudong Wang, Shipo Wu, Busen Wang, Jinlong Zhang, Xiaohong Song, Yi Chen, Peng Lv, Lihua Hou

**Affiliations:** Laboratory of Vaccine and Antibody Engineering, Beijing Institute of Biotechnology, Beijing, China

**Keywords:** immunogenicity, Ebola (EBOV), intradermal, intramuscular, Ad5-vector vaccine

## Abstract

The proper route for vaccine delivery plays an important role in activating a robust immune response. Several viral vector-based vaccines against Ebola disease administered intramuscularly have been found to have excellent immunogenicity and protectiveness. In this study, we evaluated different vaccine routes for Ad5-EBOV delivery by comparing humoral and cellular responses, germinal center reactions, dendritic cell activation and antigen expression. Mice injected intramuscularly with the vaccine exhibited an advantage in antigen expression, leading to more robust germinal center and humoral responses, while intradermal injection recruited more migrating DCs and induced a more polyfunctional cellular response. Our study provides more data for future use of viral vector-based vaccines.

## Introduction

Ebola virus (EBOV), a member of the filoviridae family, can cause severe hemorrhagic fever with high mortality up to 90%, as shown in different outbreaks (1). Since EBOV was first reported in 1976, outbreaks have occurred sporadically in rural areas in some Central African countries (2). The 2013–2016 EBOV outbreak in West Africa resulted in more than 28600 human infections and over 11300 deaths (3). Recently, several laboratory-confirmed cases were confirmed and reported by The Ministry of Health of the Democratic Republic of the Congo (4). The persistent Ebola epidemic indicates the need for further development of vaccines against Ebola diseases. Various kinds of vaccine candidates, including DNA vaccines, viral vector-based vaccines, whole-virus vaccines and viral particle vaccines, have shown robust immunogenicity in non-human primates or human studies (5). Two viral vector-based vaccines, adenovirus type 5 (Ad5)-EBOV and rVSV-EBOV-GP, induced robust immunogenicity and tolerable safety in the clinical study and were gained conditional approval by the government to prevent EBOV-caused diseases (6, 7).

Compared to traditional vaccines, viral vector-based vaccines have the main advantage of evoking robust adaptive humoral and cellular immune responses in the absence of an adjuvant (8). This kind of vaccine can maintain the characteristics of the viral vector, such as infection of host cells, and express an antigenic protein instead of producing progeny viral particles. The antigenic protein is then taken up by antigen-presenting cells, such as dendritic cells (DCs), and presented to naive CD4+ T cells in the draining lymph nodes (LNs), leading to the differentiation of T follicular helper (Tfh) cells and the generation and maintenance of germinal centers (GCs). Finally, an adaptive immune response involving humoral and cellular reactions is triggered with the aforementioned help.

The immune response elicited by a vaccine is deeply correlated with the administration route (9). Various routes of vaccine delivery have been frequently used; these include the subcutaneous, intramuscular and intranasal routes. In the past decade, intradermal administration has been thought to be a more time-saving and effective approach for delivering antigens directly to antigen-presenting cells and augmenting specific immune reactions, since the skin is one of the most immunologically active organs due to the presence of functionally diverse DC subsets (10–13). The proof of principle for immunization *via* the skin has been established since Edward Jenner introduced vaccinia virus into the skin to generate protection against smallpox in 1796 (14). Recently, several intradermally delivered vaccines, such as inactivated rabies, poliovirus and influenza vaccines, have shown immunogenicity comparable to that of intramuscularly delivered vaccines with a dose-sparing strategy (15–17). Preclinical studies on viral vector-based vaccines, including ChAd63- and rAAV2/1-based vaccines, have shown that these vaccines induce abundant T cell responses (18, 19). Increasing research on intradermal delivery has demonstrated its unique advantage in stimulating an immune response.

Our earlier study proved the induction of strong humoral and cellular responses in humans intramuscularly vaccinated with Ad5-EBOV (6, 20). Whether intradermal delivery can work as an ideal route for delivery of this vaccine has not been investigated. In this research, we compared the specific humoral and cellular immune responses, DC and GC activation, and antigen expression achieved *via* vaccine delivery by different routes. Our results revealed that both delivery routes were able to induce humoral and cellular immune responses. Intramuscular injection induced a stronger specific IgG response and had more advantages in overcoming preexisting adenovirus immunity, while intradermal injection elicited a more abundant polyfunctional T cell response. More migrating DCs (migDCs) were recruited to the LNs by intradermal injection, but the GC reaction was not obviously improved compared to that induced by intramuscular injection. Most importantly, superior production of the antigen expressed by the viral vector was observed in mice injected intramuscularly. Overall, our study showed that intramuscular and intradermal injection of Ad5-EBOV have different characteristics in eliciting the humoral and cellular immune response, providing immune data for the usage of Ad5 vector-based vaccines.

## Materials and methods

### Mice

Female Balb/c mice (6–8 weeks old) were purchased from Beijing Vital River Laboratory Animal Technology Co., Ltd. (Beijing, China) Mice were acclimated for 1 week prior to the beginning of the experiments. Mice were kept under specific pathogen-free conditions at the animal facility of the Beijing Institute of Biotechnology. All animal experimental protocols were approved by the Animal Care and Use Committee of the Beijing Institute of Biotechnology.

### Ad5-EBOV and Ad5-Luciferase

Ad5-EBOV is a recombinant Ad5 vector-based EBOV disease vaccine that we developed during the Ebola pandemic in 2014–2016. Ad5-Luciferase (Ad5-Luc) is a recombinant adenovirus expressing the luciferase protein. The Ad5-EBOV and Ad5-Luc vectors used in this study were constructed, expressed, purified, tittered and diluted to the target dose for mice in the laboratory. Viruses were quantified using infection-forming units (ifu).

### Binding antibody analysis

Specific IgGs against the EBOV glycoprotein (GP) and Ad5 hexon protein were measured by ELISA as previously described. Briefly, ELISA plates were coated with 200 ng of vaccine-matched 2014 Zaire Makona GP or Ad5 Hexon protein overnight at 4°C. The wells were blocked with Phosphate buffer saline (PBS) containing 2% Fetal Bovine Serum (FBS) and then incubated with serum samples from mice at 3-fold serial dilutions starting at 1:33 or 1:100. Binding IgG was detected with an appropriate secondary antibody (goat anti-mouse IgG-HRP [1:10000; Abcam]) and a tetramethylbenzidine (TMB) substrate solution. Antibody titer is reported as the enzyme-linked immunosorbent assay (ELISA) 90% effective concentration (EC_90_; the most dilute serum concentration at which there is a 90% decrease in antigen binding). EC_90_ values were calculated with GraphPad Prism (version 8.0.2) with subtraction of the negative serum optical density.

### T cell response assessment

Mice were sacrificed on day 14 post-vaccination, and splenocytes were isolated by pushing the spleen through a 70-μm cell strainer. After washing and counting, 2.5×10^6^ cells were stimulated for 6 h at 37°C with or without 1.2 μg/mL overlapping 15-amino-acid peptides covering the Zaire Makona GP under incubation with AF647-conjugated anti-CD107a (clone XMG1.2, 1:4000 dilution), GolgiStop (BD Biosciences, USA) and GolgiPlug (BD Biosciences, USA). After stimulation, the cells were washed and stained with a mixture of antibodies against lineage markers, including PerCP-Cy5.5-conjugated anti-CD3 (clone 17A2, 1:330 dilution), Alexa Fluor (AF) 700-conjugated anti-CD4 (clone RM4-5, 1:1000 dilution), and FITC-conjugated anti-CD8a (clone 5H10-1, 1:250 dilution), and the viability dye Near-IR to exclude dead cells from the data analysis. After one wash with PBS, the cells were fixed and permeabilized with Cytofix/Cytoperm (BD Biosciences, USA), washed with Perm/Wash buffer (BD Biosciences, USA), and stained with PE-conjugated anti-IFNγ (clone XMG1.2, 1:100 dilution), PE-Cy7-conjugated anti-TNFα (clone MP6-XT22, 1:100 dilution) and Brilliant Violet (BV) 421-conjugated anti-IL2 (clone JES6-5H4, 1:160 dilution). The cells were washed successively with Perm/Wash buffer and PBS and resuspended in PBS, and data were acquired on a FACSCanto Plus (BD Biosciences, USA). At least 300000 events were collected for each sample, and the data were analyzed with FlowJo 10 software.

### Evaluation of Tfh and GC B cell reactions

To track GC reactions, GC B cells and Tfh cells were studied by flow cytometry. Fresh inguinal and popliteal LNs were collected from sacrificed mice on days 7, 14 and 28 post-vaccinations, pooled in 500 μL of PBS and then disaggregated mechanically. The LNs were filtered through a 70-μm cell strainer and centrifuged at 600 g for 5 min. To exclude nonspecific binding signals and dead cells, cells were incubated with anti-CD16/32 antibodies and the viability dye Near-IR on ice for 30 min. After washing, an antibody cocktail for GC B cells including AF700-conjugated anti-CD4 (clone GK1.5, 1:50), PerCP-Cy5.5-conjugated anti-IgD (clone 11-26c.2a, 1:50), PE-conjugated anti-GL7 (clone Gl7, 1:25), and APC-conjugated anti-B220 (clone Ra3-6b2, 1:25) and an antibody cocktail for Tfh cells including AF700-conjugated anti-CD4 (clone GK1.5, 1:50), BV510-conjugated anti-CD3 (clone 17a2, 1:25), PE-Cy7-conjugated anti-CXCR5 (clone L138d7, 1:50), BV421-conjugated anti-PD-1 (clone 29f.1a12, 1:25), and PE-conjugated anti-CD44 (clone Sa367h8, 1:25) were separately mixed with cells and incubated on ice for 30 min. After washing and resuspension in by PBS, the samples were immediately run on a BD FACSCanto Plus analytical flow cytometer. The FCS data files were further processed using FlowJo 10 software. The gating strategy is described in the **Supplementary Figure**.

### Subtyping of migDCs in the LNs

migDCs in the LNs were subtyped using the flow cytometry. Briefly, mice were euthanized 1 day after vaccination, and the popliteal, inguinal, axillary, and brachial LNs were harvested and pooled in 1 mL of Hank’s balanced salt solution (Gibco) with 400 U/mL collagenase IV (Roche) and 10 mM EDTA, followed by incubation at 37°C for 5 min. The digested LNs were then filtered through a 70-μm cell strainer and washed with 2 mM EDTA in PBS before staining. The cells were incubated with anti-CD16/32 antibodies and the viability dye Near-IR on ice for 30 min. Further subtyping was performed with an antibody cocktail containing APC-conjugated anti-MHC-II (clone M5/114.15.2, 1:100), PE-Cy7-conjugated anti-CD11c (clone N418, 1:100), PerCP-Cy5.5-conjugated anti-CD11b (clone M1/70, 1:50), FITC-conjugated anti-CD103 (clone 2e7, 1:50), PE-conjugated anti-CD207 (clone 4c7, 1:50) and BV510-conjugated anti-CD86 (clone GL-1, 1:25). The samples were run on a BD FACSCanto Plus analytical flow cytometer and analyzed using FlowJo 10 software. The gating strategy is described in the **Supplementary Figure**.

### *In vivo* imaging

Mice were injected intramuscularly or intradermally with 10^7^ ifu of Ad5-Luc (N=5 per group). The intensity of the luciferase signal measured by bioluminescence imaging was considered to represent the expression of the antigen. Bioluminescence images were acquired and analyzed with the IVIS Spectrum *In Vivo* Imaging System (PerkinElmer Inc., Waltham, MA, USA) on days 1, 3, 7, and 14 after injection. Next, D-Luciferin Firefly (PerkinElmer, dissolved in PBS at a concentration of 15 mg/mL) was intraperitoneally injected into each mouse at a working dose of 150 mg/kg. The mice were placed into a Plexiglas anesthesia box (2.5–3.5% isoflurane) for 10 min before being transferred to the imaging chamber for *in vivo* imaging. Luminescence was measured over 5 min. The relative intensities of emitted light are presented as the average radiance in p/s/cm^2^/sr.

### Statistical analysis

All of the statistical analyses and graphing were performed using GraphPad Prism 8.0.2 software. Different vaccine groups were compared using an unpaired t test or one-way ANOVA followed by Tukey’s multiple comparison *post hoc* test. A p value <0.05 was considered significant. Details are provided in the figure legends.

## Results

### Intramuscular vaccination with Ad5-EBOV elicits a more robust humoral immune response than intradermal vaccination in mice

**Figure 1:**
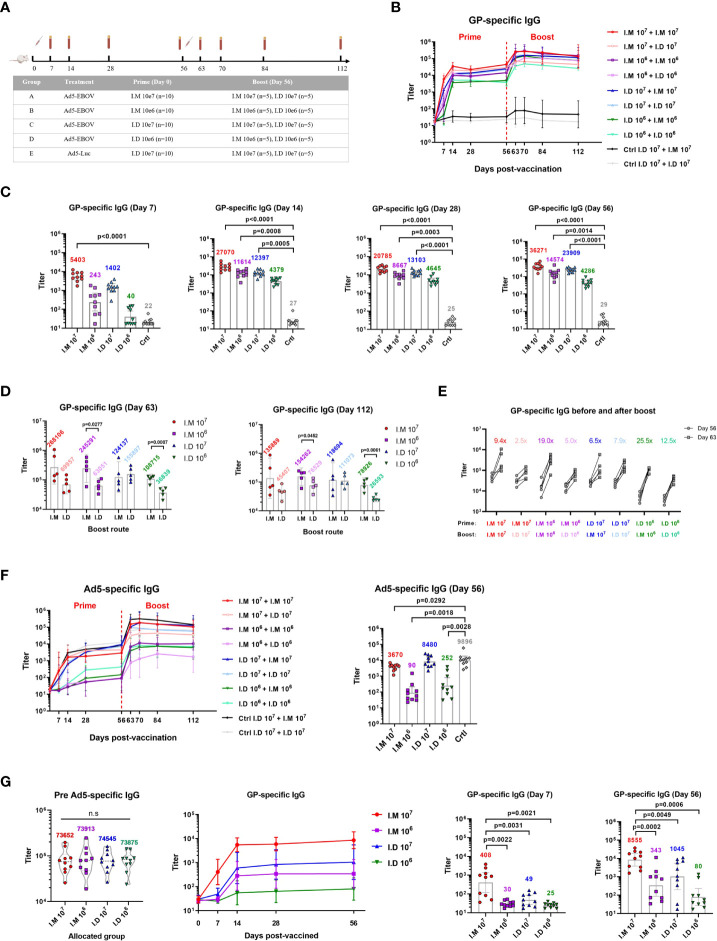
Intramuscular vaccination with Ad5-EBOV elicits a more robust humoral immune response than intradermal vaccination in mice. **(A)** BALB/C mice were primed and boosted at an interval of 8 weeks by intramuscular or intradermal routes. Blood was collected at indicated time points. **(B)** Overall GP-specific IgG response. **(C, D)** GP-specific IgG in serum was measured and compared on day 7, 14, 28 and 56 after the prime **(C)** and day 7, day 56 after the boost **(D)**. **(E)** Increase ratio (Day63/Day56) of GP-specific IgG titer was calculated after the boost. **(F)** Overall Ad5-specific IgG response was showed and compared on day 56 after the prime. **(G)** GP-specific IgG response was induced by Ad5-EBOV in mice with Ad5 pre-existing antibody. Data is represented as geometric mean with 95% CI, or mean with IQR. Statistical significance was determined using unpaired t’ test, or one-way ANOVA followed by Dunnett’s multiple comparisons test.

The humoral response is a crucial protective factor against Ebola disease. We first assessed the GP-specific IgG response induced by Ad5-EBOV administered *via* different routes. Fifty mice were intramuscularly or intradermally injected with 10^6^ or 10^7^ ifu of Ad5-EBOV or 10^7^ ifu of Ad5-Luc as a control (**Figure 1A**). Eight weeks after the initial injection, the mice in each group were equally assigned into two subgroups and boosted with the same dose of the vaccine *via* one of the two routes.

The level of GP-specific IgG was increased in a dose-dependent manner, and intramuscular injection elicited a more powerful response than intradermal injection (**Figure 1B**). The IgG titer peaked at 2-4 weeks and lasted at least 8 weeks after the priming vaccination. Among the mice in all the groups, the mice intramuscularly injected with 10^7^ ifu of Ad5-EBOV showed the best humoral response, with a superior IgG response at 7 days after vaccination compared to that of control mice (p<0.0001) (**Figure 1C**). The 10^6^ ifu intramuscular injection (p=0.0008) and 10^7^ ifu intradermal injection (p=0.0005) substantially increased the IgG level at 14 days (**Figure 1C**). The lowest IgG titer was induced by 10^6^ ifu intradermal injection and was not significantly different from the control titer. This result indicated that one injection of 10^7^ ifu of Ad5-EBOV could induce an obvious GP-specific antibody response, regardless of the route used.

All vaccinated mice showed an increase in the antibody response after the booster immunization (**Figure 1B**). Compared to intradermal injection, intramuscular injection was more advantageous for generating IgG with the booster immunization (**Figure 1D**). A similar result was not found in the mice intradermally injected with the 10^7^ ifu vaccine as the priming vaccination. Notably, a second dose of 10^6^ ifu intramuscular vaccination achieved the highest potency, with a higher level (geometric mean titer = 154262) and fold change (19.0x) for the IgG titer than those induced with the 10^7^ ifu intramuscular vaccination (geometric mean titer = 135889, fold change = 9.4x) (**Figures 1D, E**).

We next tracked the antibody response against the viral vector since viral vector-based vaccines usually causes concerns regarding disadvantages related to generating preexisting immunity. Ad5-specific IgG was also elicited in a dose-dependent manner (**Figure 1F**). Intradermal injection of Ad5-EBOV seemed to induce a higher level of IgG against Ad5 than intramuscular injection in the priming vaccination, since a notably lower level of Ad5-specific IgG was observed compared to the control level (P=0.0292) (**Figure 1F**). This might explain why a weak improvement in IgG was observed in the mice initially injected with 10^7^ ifu of Ad5-EBOV *via* the intradermal route. A mouse model with a preexisting Ad5-specific antibody repertoire was established by intranasal infection with 10^7^ ifu of Ad5. Four weeks after infection, the mice were assigned to 4 groups (N=10 per group) to create groups with equivalent geometric mean titers of Ad5-specific IgG (**Figure 1G**). Next, the mice were injected with a low or high dose of Ad5-EBOV *via* the different routes. Obviously, the titer of GP-specific IgG elicited in this mouse model was decreased to approximately one-tenth of the titer in normal mice. A significantly higher titer of GP-specific IgG was observed in only mice intramuscularly injected with 10^7^ ifu of Ad5-EBOV. Other groups generated only a weak IgG response that was not significantly different from the control response. These results further suggested that intramuscular injection of Ad5-EBOV has a stronger advantage in eliciting GP-specific IgG and overcoming preexisting immunity against Ad5.

### Intradermal injection stimulates a richer multifunctional cellular immune response than intramuscular injection

**Figure 2:**
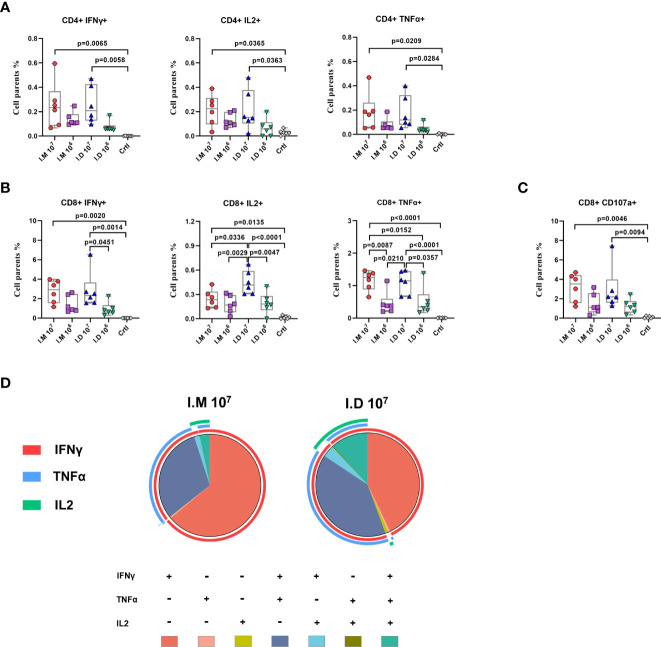
Intradermal injection stimulates a richer multifunctional cellular immune response than intramuscular injection. **(A–C)** Glycoprotein-specific cytokines (IFNγ, IL-2, TNFα) producing CD4+ T cells **(A)**, CD8+ T cells **(B)** and CD107 producing CD8+ T cells **(C)** was measured and analyzed by a flow cytometry. **(D)** Proportions of glycoprotein-specific CD8+ T cells that produce any combination of the three cytokines at day14. Data is represented as median with 95% CI. Statistical significance was determined using one-way ANOVA followed by Tukey’s multiple comparisons test.

We previously reported that Ad5-EBOV could induce a robust cellular immune response on day 14 post-vaccination. Given the superior humoral response observed with intramuscular injection, we examined the impact of the two delivery routes on vaccine-induced T cell responses. Both intramuscular injection and intradermal injection significantly activated the Ebola peptide pool-experienced CD4+ and CD8+ cellular immune responses in a dose-dependent manner (**Figures 2A, B**). A notable increase in the Th1-biased cellular response, including TNFα+, IL2+ and IFNγ+CD8+ T cells (**Figure 2B**), was found in mice that received a high dose of the vaccine, and CD107+CD8+ T cells, representing the function of degranulation, were also increased (**Figure 2C**). Notably, the IL2-producing CD8+ T cell response stimulated by high-dose intradermal injection was significantly stronger than that stimulated by intramuscular injection (p=0.0336). Similar kinetics were observed for the specific CD8+ T cell response that presented with polyfunctional phenotypes (IFNr+TNFa-IL2+ or IFNr+TNFa+IL2+) (**Figure 2D**). These results indicated that intradermal injection produced a comparably strong cellular immune response compared to intramuscular injection but with a larger multifunctional T cell population.

### Intramuscular and intradermal injections elicited similar Tfh and GC B cell responses but induced different levels of response

**Figure 3:**
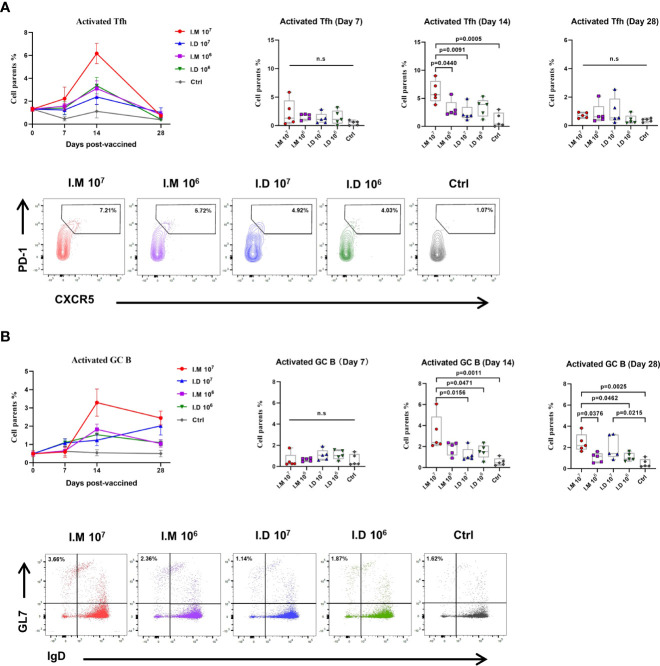
Intramuscular and intradermal injections elicited similar Tfh and GC B cell responses but induced different levels of response. Inguinal and popliteal lymph nodes were sampled at indicated times following immunization to measure the Tfh and GC B response. **(A)** CD3+CD4+PD1+CXCR5+ Tfh was determined and compared in mice injected with Ad5-EBOV in different doses and routes. **(B)** CD4+B220+GL7+IgD- GCB was determined and compared in mice injected with Ad5-EBOV in different doses and routes. Data is represented as median with 95% CI. Statistical significance was determined using one-way ANOVA followed by Tukey's multiple comparisons test. n.s indicated no significance.

Tfh and GC B cells responses are thought to be closely related to the adaptive humoral response. To further explain the humoral response, the dynamics of Tfh and GC B cell reactions were investigated by flow cytometry. Characterized LNs were disaggregated into single-cell suspensions and stained with an antibody cocktail to quantify Tfh cells and GC B cells. The proportions of GC B and Tfh cells were slightly upregulated on day 7, peaked on day 14 and gradually decreased through day 28 after vaccination (**Figures 3A, B**). No obvious activation was observed in mock-treated mice. Specifically, 10^7^ ifu intramuscular injection elicited superior responses compared to other treatments at 2 weeks post-vaccination. After 4 weeks, the level of the Tfh cell response in all groups had almost diminished to the baseline level, but the GC B cell response still appeared to be robust. However, the GC B cell response on day 28 elicited by 10^7^ ifu intradermal injection was higher than that on day 14, but this difference did not reach statistical significance because of within-group variation (**Figure 3B**). These results demonstrated that both routes induced similar Tfh and GC B cell responses and that the stronger activation induced by intramuscular injection was consistent with the GP-specific IgG levels.

### Intradermal injection modulated more migDCs but had a comparable activation level as intramuscular injection

**Figure 4:**
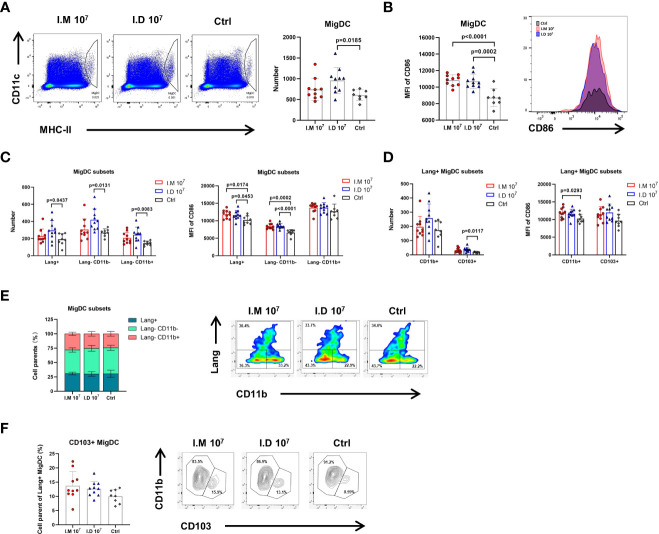
Intradermal injection modulated more migDCs but had no advantage in activation ability. Mice were killed on day 1 post-injection and popliteal, inguinal, axillary, and brachial LNs were harvested for further analysis. **(A)** Representative gates and numbers of migrate DCs in mice injected with different routes. **(B)** Representative median fluorescence intensity of CD86 expression for migrate DCs population in mice injected with different routes. **(C)** Numbers and MFI of CD86 expression for migrate DCs subsets (Lang+, Lang-CD11b-, Lang-CD11b+) was compared. **(D)** Numbers and MFI of CD86 expression for Lang+ migrate DCs subsets (CD11b+ and CD103+) was compared. **(E)** The proportions of migrate DCs subsets were compared in mice with different routes. **(F)** The proportions of Lang+ migrate DCs subsets were compared in mice with different routes. Data is represented as genomic mean ± SD. Statistical significance was determined using one-way ANOVA followed by Tukey’s multiple comparisons test.

The skin is one of most immunologically active organs due to the presence of immune cells. To further identify how antigen presentation is influenced by different immunization routes, DCs were isolated from multiple pooled LNs and subtyped at 24 h post-vaccination. Tissue migDCs were first characterized as MHC-II^hi^CD11c^int^ cells, followed by subtyping into various functionally distinct subsets including Langerin-CD11b-, Langerin-CD11b+, Langerin+CD103+CD11b-, and Langerin+CD103-CD11b+ Langerhans cells. Intradermal injection increased the number of migDC populations compared with control treatment (p=0.0185) (**Figure 4A**). However, superior expression of CD86 on migDCs was found in both the intradermal (p=0.0002) and intramuscular (p<0.0001) groups, meaning that intradermal injection barely differed from intramuscular injection in activating DCs (**Figure 4B**). Similar results were observed with further typing (**Figures 4C, D**). The mice in the intramuscular group had a marginal increase in the Langerin-CD11b+ migDC population compared with the mice in the control group (**Figure 4E**). Among all Langerin+ migDCs, both intramuscular injection and intradermal injection slightly expanded the CD103+ population but no significant different was found (**Figure 4F**). It seemed that intramuscular injection was capable of altering CD11b+Langerin- migDCs and CD103+Langerin+ migDCs. Together, these findings demonstrated that intradermal injection of Ad5-EBOV recruited more migDCs than intramuscular injection, but the levels of activation were similar.

### Intramuscular injection exhibited a prominent effect on antigen expression

**Figure 5:**
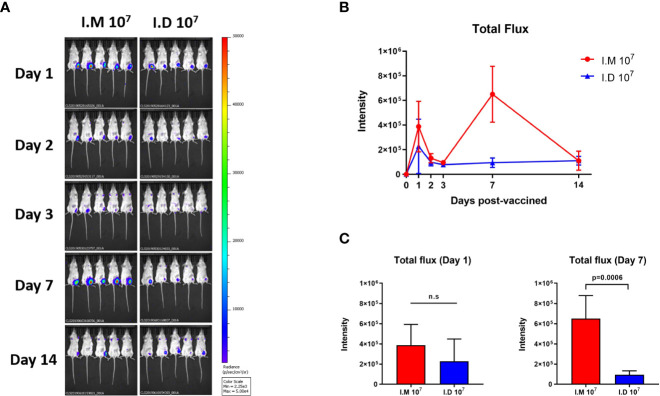
Intramuscular injection exhibited a prominent effect on antigen expression. Ad5-Luc is injected intramuscularly or intradermally and the photo flux was detected at indicated times. **(A, B)** Bioluminescent images were acquired and Total flux was calculated at day 1, 2, 3, 7 and 14 post-injections. **(C)** Values for average radiance at each time point are compared. Data is represented as mean ± SD. Statistical significance was determined using unpaired t' test. n.s indicated no significance.

We next explored the antigen expression induced by the different routes since antigen expression is a prerequisite for stimulating a robust immune response. Ad5-Luc was used for intramuscular or intradermal injection to simulate the vaccination process, and the photoflux, representing antigen expression, was detected on days 1, 2, 3, 7 and 14 post-injections. The reporter protein in the intradermal group peaked on day 1 post-injection, followed by a rapid reduction (**Figures 5A, B**). For intramuscular injection, luciferase was expressed at a higher level than that in the intradermal group, but the difference was not significant at day 1 (**Figure 5C**). Notably, another higher peak in luciferase expression was reached on day 7, which was more robust than that in the intradermal group on the same day (p=0.0006). Both delivery routes exhibited very low expression on day 14 with no differences. The data implied that intramuscular injection might produce better antigen expression than intradermal injection using a syringe for the Ad5 vector-based vaccine.

## Discussion

In general, intramuscular and subcutaneous immunizations are frequent routes for vaccine injection, the effects of which have been proven historically. Intradermal delivery imparted more immunogenic properties to inactivated and subunit vaccines because of the abundant immunostimulatory cells in the skin. However, whether the delivery route can influence the immune responses induced by viral vector-based vaccines has not been extensively tested. We previously suggested that intramuscular injection of Ad5-EBOV could invoke humoral and cellular responses in animals and humans. Based on the evidence, this study was designed to investigate the immunogenicity of intradermal delivery of Ad5-EBOV.

Antibody responses have been shown to be necessary for vaccine-mediated protection against Ebola virus. The robust EBOV GP-specific antibody response is thought to be correlated with good protectiveness (21). It has been reported that depletion of CD4+ T cells in nonhuman primates during vaccination caused a complete loss of GP-specific antibodies and abrogated vaccine protection, while the animals depleted of CD8+ T cells were survived from the attack of lethal Ebola virus (22). By comparing the induction of GP-specific IgG in adenovirus-naive mice injected with Ad5-EBOV by different routes, we found that intramuscular injection was superior to intradermal injection, eliciting stronger IgG responses with both the priming and booster immunizations. Furthermore, a dose two times higher than the low dose of Ad5-EBOV induced a very high IgG level, which was even better than that induced with the high dose. In mice with preexisting immunity to adenovirus, intramuscular injection exhibited a better ability to overcome the influence of antibodies against Ad5. There are two potential explanations. On the one hand, the intramuscular injection site usually has a large area for vaccine release and abundant blood vessels to enable extensive antigen delivery (23). In this study, mice showed a superior advantage in antigen expression with a “double-activation” mode following intramuscular delivery of Ad5-Luc, which may further affect the adaptive immune response. On the other hand, mice injected with Ad5-EBOV intradermally seemed to develop a more obvious Ad5-specific response, indicating that the GP-specific response was hampered to some extent. Other studies have demonstrated that immunization with microneedle patches instead of a syringe can effectively attenuate the immune response against the viral vector (24). To date, intramuscular delivery supported Ad5-EBOV to achieve the higher antigen expression and GP-specific humoral response.

In GCs, Tfh cells interact with B cells to support the differentiation and maturation of B cells into plasma cells, which is highly correlated with antibody generation (25). Here, we explored the Tfh and GC B cell reactions activated by Ad5-EBOV delivery *via* different routes. It has been reported that the intramuscular adenovirus vectored vaccines were potent in inducing GC B and Tfh response at day 7 after the immunization (26). In this study, the Tfh and GC B cell responses exhibited a dynamic change similar to that of the GP-specific IgG, which were appeared at week 1 post-injection and peaked at week 2 post-injection. Then the Tfh and GC B cells gradually decreased due to the attenuation of antigen expression. A stronger response in the LNs, despite the non-GP-specific response, was induced by intramuscular injection rather than intradermal injection, which is consistent with the humoral response data. Although injection into the dermis is beneficial for increasing DC subsets migrating to the LNs, the similar intensity of CD86+ on migDCs indicated an equivalent activation of antigen-presenting cells by the two delivery routes. Notably, a higher proportion of Langerin-CD11b+ migDCs, which are characterized as an important functional cell group that takes up soluble antigens (27), was induced by intramuscular injection. We hypothesized that this could be one of the reasons for the difference in the induction of a humoral response, but more in-depth studies are needed.

Quick and robust elicitation of cellular response is the distinct feature of viral vector-based vaccines, and intradermal injection further enhanced this advantage. Both the CD4+ and CD8+ T cell responses generated by intradermal vaccination reached levels comparable to those induced by intramuscular vaccination. TNFα+, IL2+ and IFNγ+ T cell populations were significantly expanded, indicating that both routes induced a Th1-dominated cellular response. Notably, intradermal delivery of Ad5-EBOV obviously improved the polyfunctional cellular immune response, especially the IL2+CD8+ T cell response. The probable mechanistic explanation may be the multiple DC subsets activated by Ad5-EBOV in the dermis (28, 29). In line with others’ findings on dermal DCs (30), our data supported the conclusion that Ad5-EBOV can recruit a larger number of DC subgroups when delivered intradermally. These outstanding results suggest that intradermal delivery can be used to expand the cellular response to viral vector-based vaccines to prevent diseases and eliminate the target virus.

In this study, two expression peaks of luciferase were found at day 1 and day 7 after a single shot of intramuscular Ad5-Luc. We hypothesized that the changes in luciferase expression were due to the innate immune response. Specifically, the intramuscular delivery of Ad5-Luc triggered the early and robust type I IFN response that impaired efficient antigen expression and antigen presentation (31, 32), thus the luciferase expression was limited and displayed a short peak at day 1. While the initial immune response receded in 3-7 days, the luciferase expression was increased again, until the adaptive immune response eliminated the host cells infected by Ad5-Luc in 1-2 weeks. We also speculated that a durability of luciferase expression was not supported by the immune environment in the dermis. Intradermal injected Ad5-Luc was eradicated quickly since the dermis contains the rich access to lymph organs and the innate immune response including macrophages and neutrophilic granulocytes.

Overall, this study described the comparable immunogenicity induced by Ad5-EBOV delivery by the intramuscular or intradermal route. Intramuscular injection of Ad5-EBOV induced a robust humoral immune response because of the stronger antigen expression and germinal center reaction, while intradermal injection elicited a more abundant polyfunctional cellular immune response by recruiting more dermal DC subsets. Due to the limitations of the ABSL-4 laboratory, the protective efficacy induced by intradermal injection of Ad5-EBOV could not be tested. Nevertheless, our findings offer immunological evidence for vaccination strategy selection for Ad5-EBOV and other viral vector-based vaccines.

## Data availability statement

The raw data supporting the conclusions of this article will be made available by the authors, without undue reservation.

## Ethics statement

The animal study was reviewed and approved by the Animal Care and Use Committee of the Beijing Institute of Biotechnology.

## Author contributions

ZZ and LH designed experiments, analyzed data and wrote the manuscript. ZH-Z and YW conducted the vaccination and sampling. ZH-Z, YW, SW and BW conducted the T cell, GC B and migDCs assessment. XS and YC finished the antibody assessment. JZ performed the *in vivo* imaging. PL conducted all the statistical analysis. All authors contributed to the article and approved the submitted version.

## Funding

The work was supported by the National Natural Science Foundation of China (project 31900671).

## Conflict of interest

The authors declare that the research was conducted in the absence of any commercial or financial relationships that could be construed as a potential conflict of interest.

## Publisher’s note

All claims expressed in this article are solely those of the authors and do not necessarily represent those of their affiliated organizations, or those of the publisher, the editors and the reviewers. Any product that may be evaluated in this article, or claim that may be made by its manufacturer, is not guaranteed or endorsed by the publisher.
