# Supplementary material for: Comparative immunogenicity analysis of intradermal versus intramuscular immunization with a recombinant human adenovirus type 5 vaccine against Ebola virus
Source: Front Immunol. 2022 Aug 31;13:963049. doi: 10.3389/fimmu.2022.963049 (PMC9472118; doi:10.3389/fimmu.2022.963049)
Supplement: Supplementary file 1 [file DataSheet_1.docx]

Supplementary Material

##
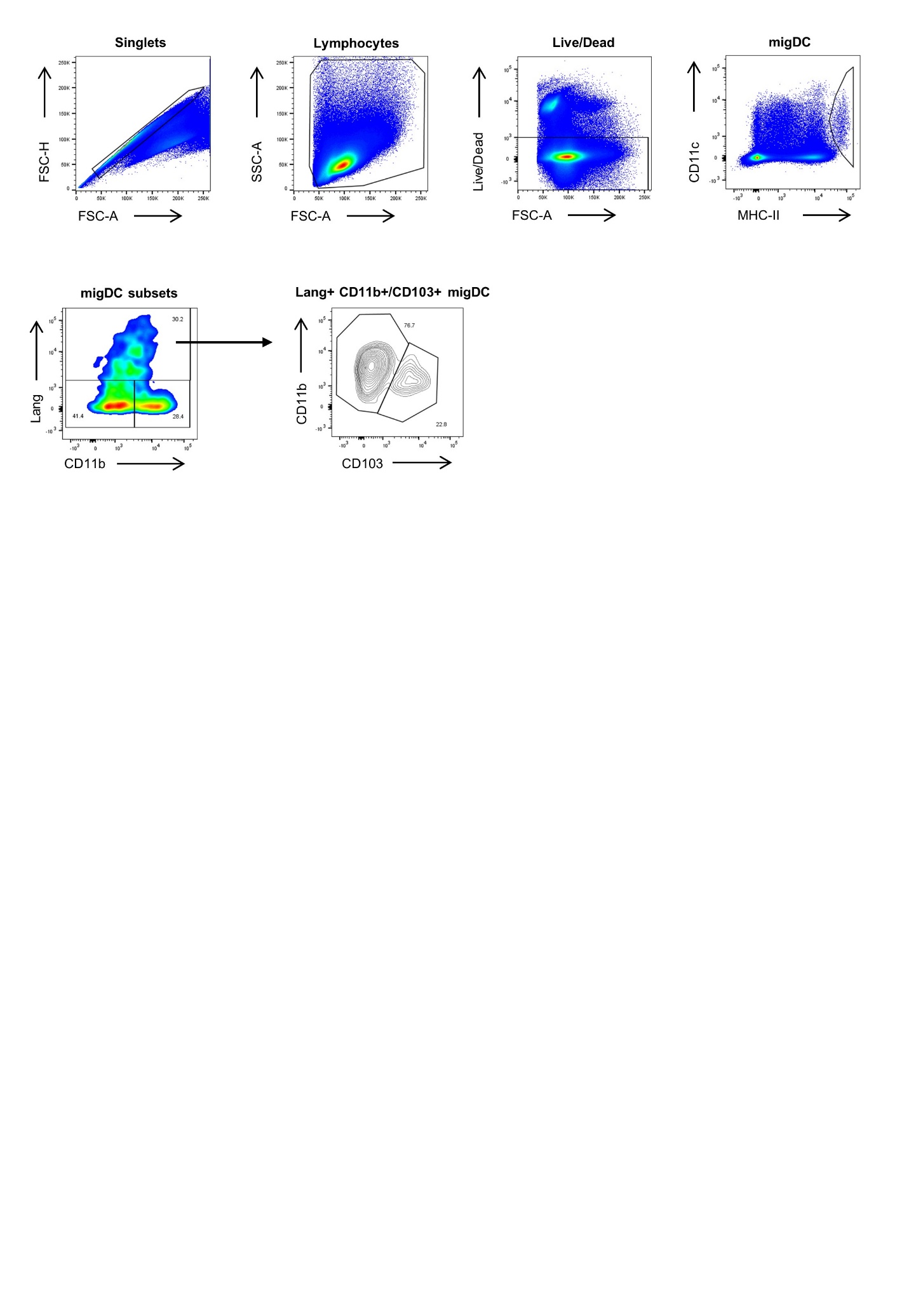
Supplementary Figures

**Supplementary Figure 1.** **Multi-parameter flow cytometry gating schematics for migrate DC cell analysis.** Gating schematic to identify migrate DC subsets within the popliteal, inguinal, axillary, and brachial lymph nodes. Dendritic cells in lymph nodes were harvested and stained for multi-parameter flow cytometry 24 hours after vaccination with intramuscular or intradermal Ad5-EBOV.

##
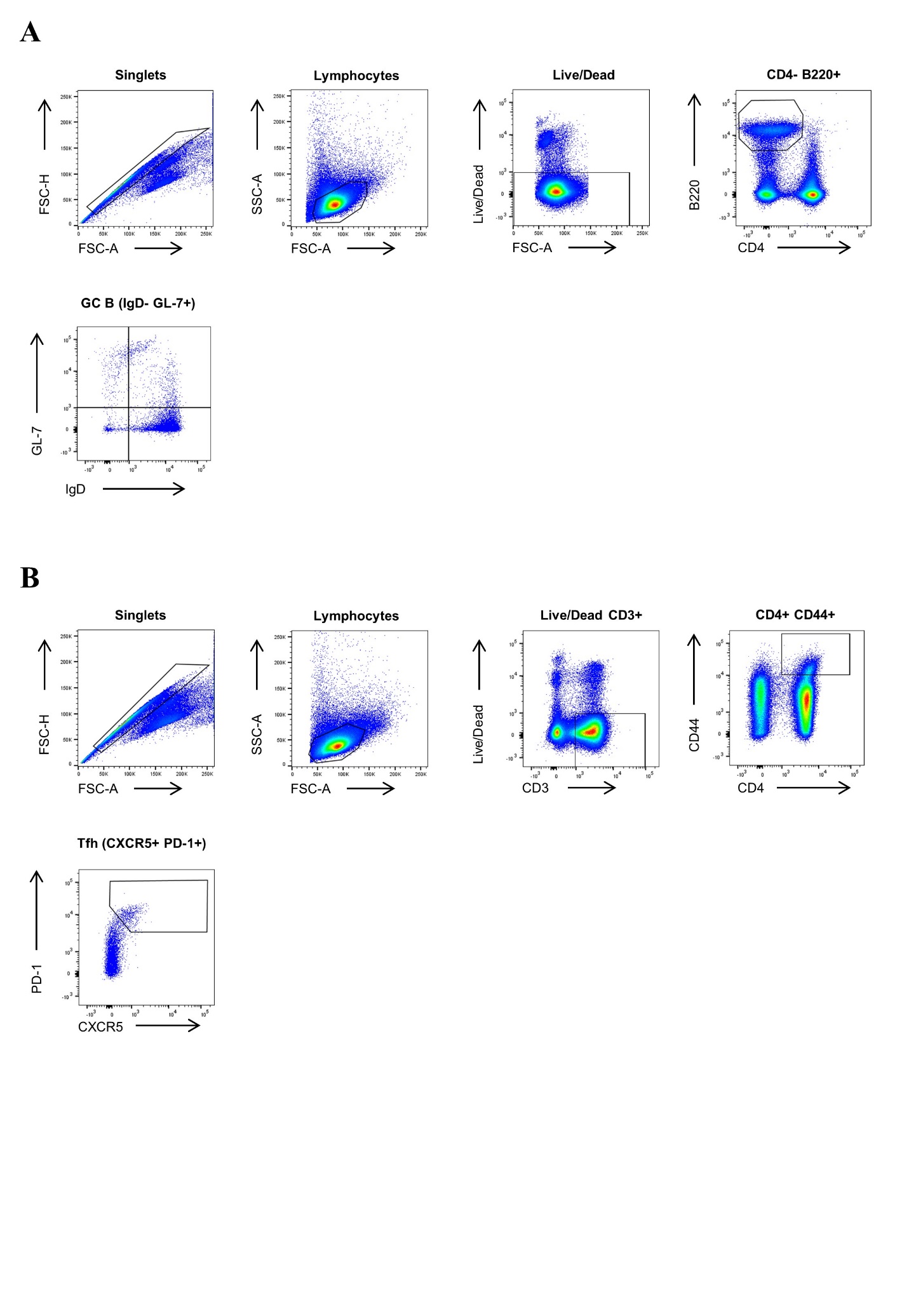


**Supplementary Figure 2.** **Multi-parameter flow cytometry gating schematics for GC B and Tfh response analysis.** Gating schematic to identify GC B and Tfh subsets within the inguinal and popliteal lymph nodes. GC B **(A)** and Tfh **(B)** cells in lymph nodes were harvested and stained for multi-parameter flow cytometry at days 7, 14 and 28 after vaccination with intramuscular or intradermal Ad5-EBOV.
